# 

**Published:** 1869-07-01

**Authors:** 


VOL. XXVI. NUMBER 13.

### THE CHICAGO Medical Journal.

PUBLISHED SEMI-MONTHLY.

J. ADAMS ALLEN, M. D., LL. D.,

WALTER HAY, A. M., M. D.,

*EDITORS.*

JULY 1st.

1869.

J. WOLCOTT HOOKER, 12 Union Building,
CHICAGO.

HORTON & LEONARD, PRINTERS, 104 AND 106 RANDOLPH STREET.

